# *N*-[6-(Di­methyl­amino)-9-phenyl-3*H*-telluro­xan­then-3-yl­idene]-*N*-methyl­methanaminium hexa­fluoro­phosphate monoclinic polymorph^1^

**DOI:** 10.1107/S2414314621005459

**Published:** 2021-06-15

**Authors:** Shea Myers, Patrick Johnson, Jason B. Benedict

**Affiliations:** a 730 Natural Sciences Complex, University at Buffalo, SUNY, Buffalo, NY 14260-3000, USA; b 771 Natural Sciences Complex, University at Buffalo, SUNY, Buffalo, NY 14260-3000, USA; University of Aberdeen, Scotland

**Keywords:** xanthene, dye, rhodamine, telluroxanthene, crystal structure, polymorph

## Abstract

The title compound C_23_H_23_N_2_Te^+^·PF_6_^−^, is a monoclinic polymorph of the previously reported triclinic structure [Calitree *et al.* (2007 ▸). *Organometallics*, **26**, 6248–6257]. In the crystal, parallel offset π–π stacking [shortest centroid–centroid separation = 3.9620 (9) Å] and ionic inter­actions help to establish the packing.

## Abstract 2

The title compound is a monoclinic polymorph of the previously reported triclinic form. In the crystal, parallel offset π-stacking and ionic inter­actions consolidate the packing.

## Graphical abstract

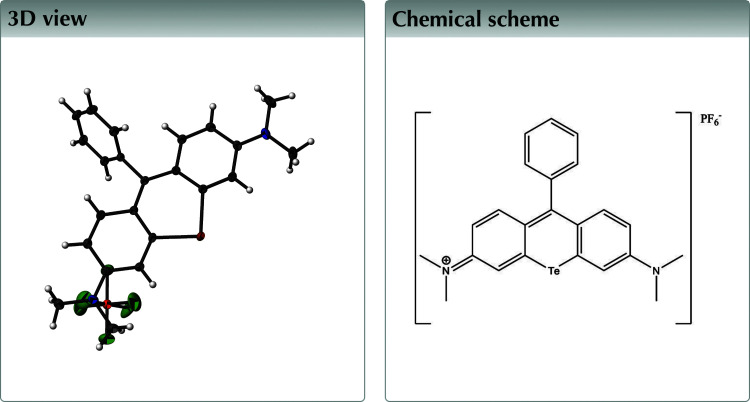

## Structure description

**Figure 1:**
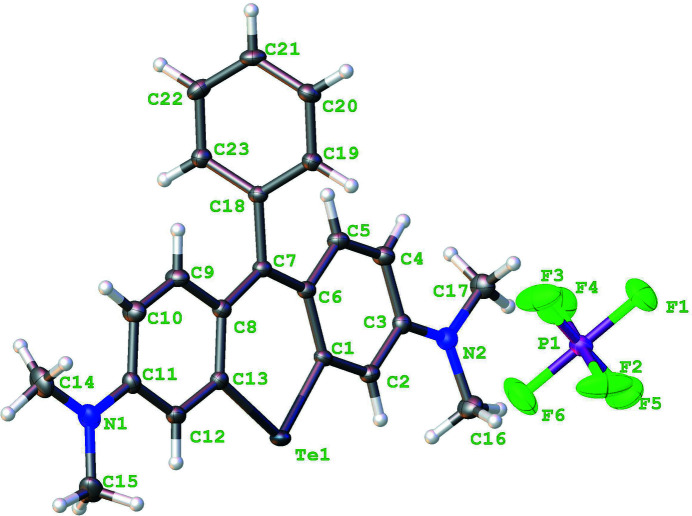
Asymmetric unit of the title compound with displacement ellipsoids drawn at 50%.

Chalcogen dyes with near-infrared absorption are used in the targeting of mitochondria of tumors (Detty *et al.*, 1990 ▸; Leonard *et al.*, 1999 ▸) and enhancing the sensitivity of medical imaging (Bedics *et al.*, 2015 ▸; Kryman *et al.*, 2016 ▸). The title compound crystallizes with a single C_23_H_23_N_2_Te^+^ telluroxanthene cation and its PF_6_^−^ counter-ion in the asymmetric unit (Fig. 1 ▸). The present monoclinic structure is a polymorph of the previously reported triclinic phase (Calitree *et al.*, 2007 ▸; Cambridge Structural Database refcode CIRPAV), which was recrystallized from the mixed solvents of aceto­nitrile and ether.

**Figure 2:**
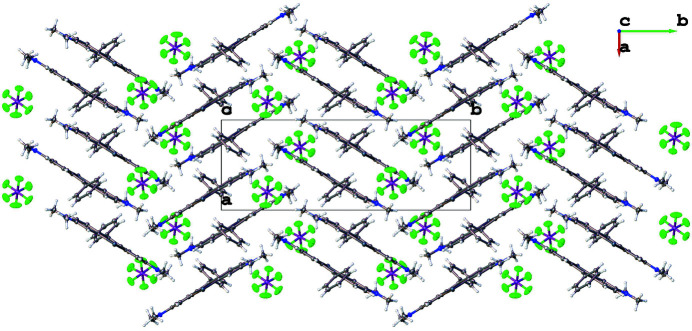
Crystal packing of the title compound viewed along [001].

**Figure 3:**
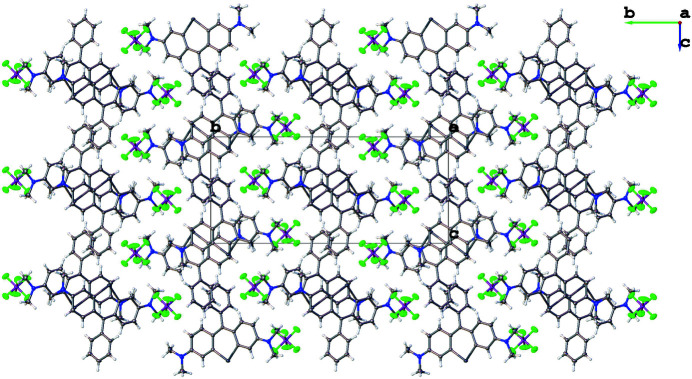
Crystal packing of the title compound viewed along [100].

The mean plane of the pendant phenyl ring (C19–C23) is nearly orthogonal to the plane of the central telluroxanthene ring (C1/C6/C7/C8/C13/Te1), which subtends a dihedral angle of 70.40 (6)°. The amine bonds (C3—N2 and C11—N1) on either side of the nearly planar telluroxanthene core (r.m.s. deviation = 0.035 Å) are almost the same length [1.343 (3) and 1.347 (3) Å, respectively] indicating delocalization of the positive charge of the cation. The crystal packing is shown in Fig. 2 ▸. The telluroxanthene cations form centrosymmetric dimer pairs, which π-stack to form columns propagating parallel to [100]. Neighboring columns inter­act along [010] to form a herringbone pattern when viewed parallel to [001] (Fig. 3 ▸).

## Synthesis and crystallization

The synthesis of title compound was previously reported (Calitree *et al.*, 2007 ▸). The title compound was dissolved in a solution of ethanol and water (70/30) and recrystallized by slow evaporation to give metallic green prisms suitable for X-ray diffraction.

## Refinement

Crystal data, data collection, and refinement details are summarized in Table 1 ▸.

## Supplementary Material

Crystal structure: contains datablock(s) I. DOI: 10.1107/S2414314621005459/hb4384sup1.cif

Structure factors: contains datablock(s) I. DOI: 10.1107/S2414314621005459/hb4384Isup2.hkl

Supporting information file. DOI: 10.1107/S2414314621005459/hb4384Isup3.cml

CCDC reference: 2085596

Additional supporting information:  crystallographic information; 3D view; checkCIF report

## Figures and Tables

**Table 1:** Experimental details

Crystal data	
Chemical formula	C_23_H_23_N_2_Te^+^·PF_6_^−^
*M*_r_	600.00
Crystal system, space group	Monoclinic, *P*2_1_/*n*
Temperature (K)	90
*a*, *b*, *c* (Å)	8.7763 (6), 23.9606 (17), 10.8738 (8)
β (°)	99.256 (2)
*V* (Å^3^)	2256.8 (3)
*Z*	4
Radiation type	Mo *K*α
μ (mm^−1^)	1.45
Crystal size (mm)	0.6 × 0.5 × 0.05

Data collection	
Diffractometer	Bruker APEXII CCD
Absorption correction	Multi-scan (*SADABS*; Bruker, 2018 ▸)
*T*_min_, *T*_max_	0.689, 0.746
No. of measured, independent and observed [*I* > 2σ(*I*)] reflections	56521, 8157, 6766
*R*_int_	0.071
(sin θ/λ)_max_ (Å^−1^)	0.767

Refinement	
*R*[*F* ^2^ > 2σ(*F* ^2^)], *wR*(*F* ^2^), *S*	0.034, 0.078, 1.02
No. of reflections	8157
No. of parameters	302
H-atom treatment	H-atom parameters constrained
Δρ_max_, Δρ_min_ (e Å^−3^)	0.67, −0.59

Computer programs: *APEX3* and *SAINT* (Bruker, 2018 ▸), *SHELXT2014/5* (Sheldrick, 2015*a* ▸), *SHELXL* (Sheldrick, 2015*b* ▸), and *OLEX2* (Dolomanov *et al.*, 2009 ▸).

## full crystallographic data

### Crystal data

**Table:**

C_23_H_23_N_2_Te^+^·PF_6_^−^	*F*(000) = 1184
*M**_r_* = 600.00	*D*_x_ = 1.766 Mg m^−^^3^
Monoclinic, *P*2_1_/*n*	Mo *K*α radiation, λ = 0.71073 Å
*a* = 8.7763 (6) Å	Cell parameters from 9937 reflections
*b* = 23.9606 (17) Å	θ = 2.5–30.3°
*c* = 10.8738 (8) Å	µ = 1.45 mm^−^^1^
β = 99.256 (2)°	*T* = 90 K
*V* = 2256.8 (3) Å^3^	Plate, metallic green
*Z* = 4	0.6 × 0.5 × 0.05 mm

### Data collection

**Table:**

Bruker APEXII CCD diffractometer	6766 reflections with *I* > 2σ(*I*)
φ and ω scans	*R*_int_ = 0.071
Absorption correction: multi-scan (SADABS; Bruker, 2018)	θ_max_ = 33.0°, θ_min_ = 1.7°
*T*_min_ = 0.689, *T*_max_ = 0.746	*h* = −13→12
56521 measured reflections	*k* = −36→36
8157 independent reflections	*l* = −16→16

### Refinement

**Table:**

Refinement on *F*^2^	Primary atom site location: dual
Least-squares matrix: full	Hydrogen site location: inferred from neighbouring sites
*R*[*F*^2^ > 2σ(*F*^2^)] = 0.034	H-atom parameters constrained
*wR*(*F*^2^) = 0.078	*w* = 1/[σ^2^(*F*_o_^2^) + (0.0237*P*)^2^ + 2.0434*P*] where *P* = (*F*_o_^2^ + 2*F*_c_^2^)/3
*S* = 1.01	(Δ/σ)_max_ = 0.005
8157 reflections	Δρ_max_ = 0.67 e Å^−^^3^
302 parameters	Δρ_min_ = −0.59 e Å^−^^3^
0 restraints	

### Special details

**Table:**

Geometry. All esds (except the esd in the dihedral angle between two l.s. planes) are estimated using the full covariance matrix. The cell esds are taken into account individually in the estimation of esds in distances, angles and torsion angles; correlations between esds in cell parameters are only used when they are defined by crystal symmetry. An approximate (isotropic) treatment of cell esds is used for estimating esds involving l.s. planes.

### Fractional atomic coordinates and isotropic or equivalent isotropic displacement parameters (Å^2^)

**Table:**

	*x*	*y*	*z*	*U*_iso_*/*U*_eq_	
Te1	0.37770 (2)	0.57306 (2)	0.60576 (2)	0.01836 (4)	
P1	0.20110 (7)	0.81612 (2)	0.40997 (5)	0.02187 (11)	
F3	0.1105 (2)	0.77039 (8)	0.32013 (17)	0.0596 (6)	
F4	0.3481 (2)	0.80903 (9)	0.34268 (19)	0.0567 (5)	
N2	0.6606 (2)	0.74434 (7)	0.42304 (17)	0.0210 (3)	
F5	0.2897 (2)	0.86218 (8)	0.49805 (18)	0.0576 (5)	
F2	0.0542 (2)	0.82231 (11)	0.47618 (17)	0.0693 (7)	
F1	0.1372 (3)	0.86213 (8)	0.30973 (18)	0.0595 (5)	
F6	0.2649 (3)	0.77015 (8)	0.50973 (19)	0.0644 (6)	
N1	0.0763 (2)	0.37834 (8)	0.56622 (18)	0.0248 (4)	
C13	0.2664 (2)	0.50923 (8)	0.49838 (18)	0.0164 (3)	
C7	0.3079 (2)	0.55013 (8)	0.29042 (18)	0.0156 (3)	
C1	0.4416 (2)	0.61632 (8)	0.45709 (17)	0.0156 (3)	
C6	0.4000 (2)	0.59734 (8)	0.33084 (18)	0.0155 (3)	
C5	0.4606 (2)	0.63079 (8)	0.24046 (19)	0.0189 (4)	
H5	0.441068	0.619248	0.155810	0.023*	
C12	0.2101 (2)	0.46640 (8)	0.56335 (19)	0.0191 (4)	
H12	0.225645	0.467975	0.651777	0.023*	
C11	0.1301 (2)	0.42024 (8)	0.5026 (2)	0.0190 (4)	
C19	0.1629 (2)	0.57900 (8)	0.08336 (19)	0.0188 (4)	
H19	0.119627	0.608620	0.124457	0.023*	
C18	0.2679 (2)	0.54247 (8)	0.15202 (17)	0.0154 (3)	
C4	0.5441 (2)	0.67792 (9)	0.26909 (19)	0.0199 (4)	
H4	0.580399	0.698301	0.204645	0.024*	
C3	0.5786 (2)	0.69747 (8)	0.39471 (19)	0.0171 (4)	
C9	0.1639 (2)	0.46274 (9)	0.30664 (19)	0.0207 (4)	
H9	0.145827	0.461284	0.218177	0.025*	
C8	0.2473 (2)	0.50933 (8)	0.36550 (18)	0.0163 (3)	
C2	0.5249 (2)	0.66452 (8)	0.48724 (19)	0.0186 (4)	
H2	0.546799	0.675927	0.571915	0.022*	
C10	0.1088 (3)	0.42026 (9)	0.3704 (2)	0.0231 (4)	
H10	0.055233	0.390205	0.325641	0.028*	
C20	0.1214 (3)	0.57237 (9)	−0.04422 (19)	0.0212 (4)	
H20	0.049254	0.597224	−0.090058	0.025*	
C23	0.3309 (2)	0.49957 (9)	0.08981 (19)	0.0195 (4)	
H23	0.402725	0.474487	0.135230	0.023*	
C22	0.2894 (3)	0.49323 (9)	−0.0381 (2)	0.0229 (4)	
H22	0.332861	0.463846	−0.079840	0.027*	
C17	0.7068 (3)	0.77967 (9)	0.3255 (2)	0.0259 (4)	
H17A	0.758035	0.756767	0.269628	0.039*	
H17B	0.778070	0.808576	0.363729	0.039*	
H17C	0.615117	0.797316	0.277811	0.039*	
C16	0.6824 (3)	0.76688 (10)	0.5492 (2)	0.0278 (5)	
H16A	0.582395	0.778526	0.569750	0.042*	
H16B	0.751810	0.799123	0.554501	0.042*	
H16C	0.727487	0.738145	0.608176	0.042*	
C21	0.1849 (3)	0.52966 (9)	−0.10492 (19)	0.0224 (4)	
H21	0.156722	0.525308	−0.192469	0.027*	
C15	0.0866 (3)	0.38130 (10)	0.7010 (2)	0.0307 (5)	
H15A	0.195362	0.381323	0.739952	0.046*	
H15B	0.034410	0.348939	0.730416	0.046*	
H15C	0.036942	0.415640	0.723435	0.046*	
C14	0.0003 (3)	0.32985 (10)	0.5022 (3)	0.0327 (5)	
H14A	−0.104010	0.340189	0.462121	0.049*	
H14B	−0.006029	0.299921	0.562561	0.049*	
H14C	0.059824	0.316883	0.438780	0.049*	

### Atomic displacement parameters (Å^2^)

**Table:**

	*U*^11^	*U*^22^	*U*^33^	*U*^12^	*U*^13^	*U*^23^
Te1	0.02524 (7)	0.01836 (7)	0.01140 (6)	−0.00151 (5)	0.00277 (5)	0.00012 (4)
P1	0.0231 (3)	0.0232 (3)	0.0192 (3)	−0.0027 (2)	0.0032 (2)	0.0013 (2)
F3	0.0821 (14)	0.0652 (12)	0.0340 (9)	−0.0458 (11)	0.0166 (9)	−0.0173 (9)
F4	0.0367 (9)	0.0773 (14)	0.0610 (12)	0.0056 (9)	0.0226 (9)	−0.0010 (10)
N2	0.0213 (8)	0.0186 (8)	0.0219 (9)	−0.0039 (6)	0.0003 (7)	−0.0002 (7)
F5	0.0738 (13)	0.0491 (11)	0.0467 (11)	−0.0247 (10)	−0.0002 (10)	−0.0150 (9)
F2	0.0339 (9)	0.146 (2)	0.0308 (9)	−0.0007 (11)	0.0147 (7)	−0.0149 (11)
F1	0.0782 (14)	0.0536 (11)	0.0438 (10)	0.0231 (10)	0.0010 (10)	0.0201 (9)
F6	0.0841 (15)	0.0490 (11)	0.0559 (12)	0.0037 (10)	−0.0014 (11)	0.0296 (9)
N1	0.0276 (9)	0.0207 (8)	0.0274 (9)	−0.0028 (7)	0.0082 (8)	0.0048 (7)
C13	0.0176 (8)	0.0161 (8)	0.0159 (8)	0.0018 (7)	0.0036 (7)	−0.0004 (7)
C7	0.0162 (8)	0.0173 (8)	0.0130 (8)	0.0011 (7)	0.0015 (7)	0.0000 (7)
C1	0.0171 (8)	0.0174 (8)	0.0123 (8)	0.0022 (7)	0.0026 (7)	0.0021 (6)
C6	0.0154 (8)	0.0172 (8)	0.0136 (8)	0.0009 (7)	0.0016 (7)	0.0003 (7)
C5	0.0201 (9)	0.0219 (9)	0.0150 (9)	−0.0005 (7)	0.0041 (7)	−0.0002 (7)
C12	0.0235 (9)	0.0189 (9)	0.0156 (9)	0.0030 (7)	0.0052 (7)	0.0013 (7)
C11	0.0198 (9)	0.0166 (9)	0.0211 (9)	0.0011 (7)	0.0050 (7)	0.0030 (7)
C19	0.0217 (9)	0.0191 (9)	0.0157 (9)	0.0019 (7)	0.0030 (7)	0.0011 (7)
C18	0.0169 (8)	0.0164 (8)	0.0130 (8)	−0.0024 (7)	0.0024 (7)	0.0002 (6)
C4	0.0212 (9)	0.0218 (9)	0.0166 (9)	−0.0026 (7)	0.0032 (7)	0.0011 (7)
C3	0.0156 (8)	0.0166 (8)	0.0186 (9)	0.0010 (7)	0.0016 (7)	0.0003 (7)
C9	0.0233 (10)	0.0236 (10)	0.0146 (9)	−0.0043 (8)	0.0010 (7)	−0.0005 (7)
C8	0.0167 (8)	0.0166 (8)	0.0155 (8)	0.0004 (7)	0.0019 (7)	0.0005 (7)
C2	0.0223 (9)	0.0181 (9)	0.0149 (8)	0.0000 (7)	0.0018 (7)	−0.0011 (7)
C10	0.0268 (11)	0.0212 (10)	0.0209 (10)	−0.0062 (8)	0.0024 (8)	−0.0010 (8)
C20	0.0225 (10)	0.0251 (10)	0.0156 (9)	0.0014 (8)	0.0018 (7)	0.0031 (7)
C23	0.0207 (9)	0.0198 (9)	0.0179 (9)	0.0024 (7)	0.0029 (7)	0.0000 (7)
C22	0.0241 (10)	0.0252 (10)	0.0199 (10)	0.0004 (8)	0.0052 (8)	−0.0069 (8)
C17	0.0277 (11)	0.0231 (10)	0.0272 (11)	−0.0077 (8)	0.0056 (9)	0.0013 (9)
C16	0.0341 (12)	0.0243 (10)	0.0239 (11)	−0.0074 (9)	0.0012 (9)	−0.0056 (8)
C21	0.0242 (10)	0.0304 (11)	0.0125 (9)	−0.0024 (8)	0.0022 (7)	−0.0024 (8)
C15	0.0397 (13)	0.0275 (11)	0.0291 (12)	0.0046 (10)	0.0188 (10)	0.0083 (9)
C14	0.0342 (12)	0.0215 (10)	0.0410 (14)	−0.0079 (9)	0.0015 (11)	0.0078 (10)

### Geometric parameters (Å, º)

**Table:**

Te1—C13	2.0711 (19)	C19—C20	1.386 (3)
Te1—C1	2.0723 (19)	C18—C23	1.393 (3)
P1—F3	1.5930 (17)	C4—H4	0.9500
P1—F4	1.5911 (18)	C4—C3	1.430 (3)
P1—F5	1.5813 (17)	C3—C2	1.418 (3)
P1—F2	1.5816 (18)	C9—H9	0.9500
P1—F1	1.5877 (17)	C9—C8	1.429 (3)
P1—F6	1.5838 (18)	C9—C10	1.363 (3)
N2—C3	1.343 (3)	C2—H2	0.9500
N2—C17	1.464 (3)	C10—H10	0.9500
N2—C16	1.458 (3)	C20—H20	0.9500
N1—C11	1.347 (3)	C20—C21	1.382 (3)
N1—C15	1.456 (3)	C23—H23	0.9500
N1—C14	1.460 (3)	C23—C22	1.388 (3)
C13—C12	1.382 (3)	C22—H22	0.9500
C13—C8	1.428 (3)	C22—C21	1.385 (3)
C7—C6	1.419 (3)	C17—H17A	0.9800
C7—C18	1.501 (3)	C17—H17B	0.9800
C7—C8	1.430 (3)	C17—H17C	0.9800
C1—C6	1.436 (3)	C16—H16A	0.9800
C1—C2	1.378 (3)	C16—H16B	0.9800
C6—C5	1.435 (3)	C16—H16C	0.9800
C5—H5	0.9500	C21—H21	0.9500
C5—C4	1.355 (3)	C15—H15A	0.9800
C12—H12	0.9500	C15—H15B	0.9800
C12—C11	1.416 (3)	C15—H15C	0.9800
C11—C10	1.419 (3)	C14—H14A	0.9800
C19—H19	0.9500	C14—H14B	0.9800
C19—C18	1.397 (3)	C14—H14C	0.9800
			
C13—Te1—C1	95.27 (8)	N2—C3—C4	121.10 (19)
F4—P1—F3	90.36 (11)	N2—C3—C2	122.01 (18)
F5—P1—F3	179.21 (12)	C2—C3—C4	116.88 (18)
F5—P1—F4	89.82 (11)	C8—C9—H9	118.2
F5—P1—F2	90.94 (12)	C10—C9—H9	118.2
F5—P1—F1	90.93 (11)	C10—C9—C8	123.63 (19)
F5—P1—F6	89.14 (11)	C13—C8—C7	125.81 (18)
F2—P1—F3	88.88 (11)	C13—C8—C9	114.86 (17)
F2—P1—F4	179.18 (13)	C9—C8—C7	119.29 (18)
F2—P1—F1	91.36 (12)	C1—C2—C3	121.47 (18)
F2—P1—F6	88.73 (12)	C1—C2—H2	119.3
F1—P1—F3	88.30 (12)	C3—C2—H2	119.3
F1—P1—F4	88.93 (11)	C11—C10—H10	119.5
F6—P1—F3	91.63 (12)	C9—C10—C11	121.0 (2)
F6—P1—F4	90.98 (12)	C9—C10—H10	119.5
F6—P1—F1	179.88 (14)	C19—C20—H20	119.9
C3—N2—C17	121.19 (18)	C21—C20—C19	120.11 (19)
C3—N2—C16	120.66 (18)	C21—C20—H20	119.9
C16—N2—C17	117.25 (17)	C18—C23—H23	119.8
C11—N1—C15	120.73 (19)	C22—C23—C18	120.46 (19)
C11—N1—C14	121.3 (2)	C22—C23—H23	119.8
C15—N1—C14	117.96 (19)	C23—C22—H22	120.0
C12—C13—Te1	115.80 (14)	C21—C22—C23	120.09 (19)
C12—C13—C8	121.69 (18)	C21—C22—H22	120.0
C8—C13—Te1	122.50 (14)	N2—C17—H17A	109.5
C6—C7—C18	115.97 (16)	N2—C17—H17B	109.5
C6—C7—C8	127.88 (18)	N2—C17—H17C	109.5
C8—C7—C18	116.15 (17)	H17A—C17—H17B	109.5
C6—C1—Te1	121.88 (14)	H17A—C17—H17C	109.5
C2—C1—Te1	115.68 (14)	H17B—C17—H17C	109.5
C2—C1—C6	122.44 (18)	N2—C16—H16A	109.5
C7—C6—C1	126.39 (17)	N2—C16—H16B	109.5
C7—C6—C5	119.26 (17)	N2—C16—H16C	109.5
C5—C6—C1	114.35 (17)	H16A—C16—H16B	109.5
C6—C5—H5	118.2	H16A—C16—H16C	109.5
C4—C5—C6	123.68 (19)	H16B—C16—H16C	109.5
C4—C5—H5	118.2	C20—C21—C22	120.00 (19)
C13—C12—H12	118.9	C20—C21—H21	120.0
C13—C12—C11	122.21 (19)	C22—C21—H21	120.0
C11—C12—H12	118.9	N1—C15—H15A	109.5
N1—C11—C12	122.1 (2)	N1—C15—H15B	109.5
N1—C11—C10	121.37 (19)	N1—C15—H15C	109.5
C12—C11—C10	116.57 (18)	H15A—C15—H15B	109.5
C18—C19—H19	119.7	H15A—C15—H15C	109.5
C20—C19—H19	119.7	H15B—C15—H15C	109.5
C20—C19—C18	120.53 (19)	N1—C14—H14A	109.5
C19—C18—C7	119.16 (17)	N1—C14—H14B	109.5
C23—C18—C7	122.02 (17)	N1—C14—H14C	109.5
C23—C18—C19	118.82 (18)	H14A—C14—H14B	109.5
C5—C4—H4	119.5	H14A—C14—H14C	109.5
C5—C4—C3	121.07 (19)	H14B—C14—H14C	109.5
C3—C4—H4	119.5		
			
Te1—C13—C12—C11	179.33 (15)	C18—C7—C6—C5	−6.0 (3)
Te1—C13—C8—C7	3.9 (3)	C18—C7—C8—C13	−177.95 (18)
Te1—C13—C8—C9	−178.28 (14)	C18—C7—C8—C9	4.3 (3)
Te1—C1—C6—C7	3.0 (3)	C18—C19—C20—C21	−0.5 (3)
Te1—C1—C6—C5	−176.93 (13)	C18—C23—C22—C21	0.0 (3)
Te1—C1—C2—C3	178.68 (15)	C4—C3—C2—C1	−0.9 (3)
N2—C3—C2—C1	−179.61 (19)	C8—C13—C12—C11	−0.2 (3)
N1—C11—C10—C9	−179.8 (2)	C8—C7—C6—C1	−5.3 (3)
C13—C12—C11—N1	179.5 (2)	C8—C7—C6—C5	174.64 (19)
C13—C12—C11—C10	−0.6 (3)	C8—C7—C18—C19	108.2 (2)
C7—C6—C5—C4	177.11 (19)	C8—C7—C18—C23	−71.0 (2)
C7—C18—C23—C22	179.00 (19)	C8—C9—C10—C11	0.8 (3)
C1—C6—C5—C4	−3.0 (3)	C2—C1—C6—C7	−176.38 (19)
C6—C7—C18—C19	−71.2 (2)	C2—C1—C6—C5	3.7 (3)
C6—C7—C18—C23	109.6 (2)	C10—C9—C8—C13	−1.5 (3)
C6—C7—C8—C13	1.4 (3)	C10—C9—C8—C7	176.5 (2)
C6—C7—C8—C9	−176.38 (19)	C20—C19—C18—C7	−178.77 (19)
C6—C1—C2—C3	−1.9 (3)	C20—C19—C18—C23	0.5 (3)
C6—C5—C4—C3	0.4 (3)	C23—C22—C21—C20	−0.1 (3)
C5—C4—C3—N2	−179.61 (19)	C17—N2—C3—C4	5.2 (3)
C5—C4—C3—C2	1.6 (3)	C17—N2—C3—C2	−176.13 (19)
C12—C13—C8—C7	−176.66 (19)	C16—N2—C3—C4	174.0 (2)
C12—C13—C8—C9	1.2 (3)	C16—N2—C3—C2	−7.3 (3)
C12—C11—C10—C9	0.3 (3)	C15—N1—C11—C12	5.5 (3)
C19—C18—C23—C22	−0.2 (3)	C15—N1—C11—C10	−174.4 (2)
C19—C20—C21—C22	0.4 (3)	C14—N1—C11—C12	−177.2 (2)
C18—C7—C6—C1	174.05 (18)	C14—N1—C11—C10	3.0 (3)
